# The Basally Expressed p53-Mediated Homeostatic Function

**DOI:** 10.3389/fcell.2021.775312

**Published:** 2021-11-23

**Authors:** Isha Nagpal, Zhi-Min Yuan

**Affiliations:** John B. Little Center for Radiation Sciences, Harvard T. H. Chan School of Public Health, Boston, MA, United States

**Keywords:** basal p53, homeostasis, metabolism, tumor suppression, p53-mediated barrier

## Abstract

Apart from mutations in the *p53* gene, p53 functions can be alternatively compromised by a decrease in nuclear p53 protein levels or activities. In accordance, enhanced p53 protein turnover due to elevated expression of the critical p53 E3 ligase MDM2 or MDM2/MDMX is found in many human cancers. Likewise, the HPV viral E6 protein-mediated p53 degradation critically contributes to the tumorigenesis of cervical cancer. In addition, growth-promoting signaling-induced cell proliferation is accompanied by p53 downregulation. Animal studies have also shown that loss of p53 is essential for oncogenes to drive malignant transformation. The close association between p53 downregulation and carcinogenesis implicates a critical role of basally expressed p53. In accordance, available evidence indicates that a reduced level of basal p53 is usually associated with disruption of homeostasis, suggesting a homeostatic function mediated by basal p53. However, basally expressed p53 under non-stress conditions is maintained at a relatively low abundance with little transcriptional activity, raising the question of how basal p53 could protect homeostasis. In this review, we summarize the findings pertinent to basal p53-mediated activities in the hope of developing a model in which basally expressed p53 functions as a barrier to anabolic metabolism to preserve homeostasis. Future investigation is necessary to characterize basal p53 functionally and to obtain an improved understanding of p53 homeostatic function, which would offer novel insight into the role of p53 in tumor suppression.

## Introduction

The function of p53 is universally disrupted in human cancers, either by a mutation in the *p53* gene locus or aberration in p53 regulation (Levine, 2020). Approximately 50% of all human cancers lost p53 function due to gene mutations, which occur primarily within the p53 DNA binding domain, underscoring the necessity of p53 binding to DNA for its tumor suppressor function (Vousden and Lane, 2007). In this context, p53 is best characterized as a transcription factor. Upon activation, p53 induces the expression of a host of genes that govern diverse cellular processes such as cell cycle progression, apoptosis, cell differentiation, and senescence, among others (Vousden and Prives, 2009), including regulation of cellular metabolic pathways. For example, studies have revealed that p53 can stimulate the mitochondrial TCA cycle by inducing the expression of SCO2 (synthesis of cytochrome oxidase 2), a critical regulator of the cytochrome c oxidase complex (Matoba et al., 2006), whereas suppressing glycolysis by repressing the expression of glucose transporters 1 and 4 (GLUT-1 & 4) (Schwartzenberg-Bar-Yoseph et al., 2004). In addition, p53 can transcriptionally induce the expression of the fructose-2,6-bisphosphatase TIGAR (p53 induced glycolysis and apoptosis regulator) (Bensaad et al., 2006). Together, available information indicates that p53 directs cellular metabolism away from glycolysis and towards oxidative phosphorylation (Vousden and Ryan, 2009). However, recent studies revealed that many p53 transcription-mediated canonic activities are dispensable for its tumor suppression (Kastenhuber and Lowe, 2017). In addition, acute DNA damage-induced p53 transcriptional activity was also found expendable for inhibition of carcinogenesis (Christophorou et al., 2006). Thus, the available information suggests that there might be unrecognized activities mediated by p53 DNA binding critical for tumor suppression.

Apart from *p53* gene mutations, the activity of p53 can be attenuated by a reduction in nuclear p53 levels (Kastenhuber and Lowe, 2017). Indeed, diminished nuclear p53 protein abundance due to overexpression of the critical p53 E3 ligase MDM2 or MDM2/MDMX is found in many human cancers (Karni-Schmidt et al., 2016). Likewise, the HPV viral E6 protein-mediated p53 degradation critically contributes to the development of cervical cancer (Scheffner et al., 1990). Thus, preclinic as well as clinical studies suggest that the amount of basally expressed nuclear p53 and its DNA sequence-specific binding are critical for p53’s tumor-suppressive function.

## The Regulation of p53

Because of its growth inhibitory activity, p53 is normally maintained at a relatively low level under physiological conditions (Vousden and Prives, 2009). Ample evidence indicates that p53 is primarily regulated, mainly at the post-translation level via protein turnover. Among many proteins involved in the regulation of p53 turnover, MDM2 stands out as the dominant E3 ligase specifically targeting p53 for ubiquitination and subsequent proteasome-dependent degradation (Haupt et al., 1997; Ringshausen et al., 2006). While MDMX, the structural homolog of MDM2, lacks intrinsic E3 ligase activity, it can modulate MDM2 E3 ligase activity via forming the MDM2/MDMX complex (Linares et al., 2003; Kawai et al., 2007). Genetic studies have provided convincing evidence demonstrating that MDM2 and MDMX are two essential negative regulators of p53, and the formation of the MDM2/MDMX complex appears crucial in p53 control (Parant et al., 2001; Huang et al., 2011). p53, MDMX, and MDM2 form a highly dynamic regulatory core that comprises positive and negative feedback loops^,^ which ensure tight regulation of p53 in non-stress conditions and its swift response to stress conditions (Gu et al., 2002). As the critical upstream modulator of p53, the MDM2/MDMX complex integrates myriad intrinsic and external signals to regulate p53 response to the perturbation of homeostasis (Wade et al., 2013)*.* In line with a protein containing the nuclear localization sequence (NLS), MDM2 is primarily nuclear-localized. Of note, despite sharing a high degree of structural similarity with MDM2, MDMX lacks the NLS and is a predominantly cytoplasmic protein (Gu et al., 2002). MDMX, however, can translocate into the nucleus upon binding to and forming a complex with MDM2. Given its cytoplasmic distribution, it is conceivable that MDMX serves as the sentinel for various signaling cues directed towards the MDM2/MDMX complex and aimed at either suppressing or activating p53 (Shadfan et al., 2012; Wang et al., 2020). Studies have shown that in response to growth-promoting signals, many mitogenic protein kinases can inhibit p53 activation via enhancing MDM2/MDMX stability and, specifically, through post-translational modifications of MDMX (Lopez-Pajares et al., 2008; Gerarduzzi et al., 2016).

Apart from MDM2, several additional E3 ligases were reported to promote p53 for ubiquitinate/proteasome degradation, including Pirh2, Cop1, TRIM proteins CHIP, RBCK1, and ARF-BP1, among others (Sane and Rezvani, 2017). Evidence suggests that while important, these E3 ligases may regulate p53 turnover in a context-dependent manner. For instance, Cop1 is amplified in certain human cancers such as hepatocellular carcinomas and breast cancer where p53 is not frequently mutated, suggesting an essential role of Cop1 in p53 inhibition in the context of these types of human cancers.

In line with the general feature of the two-directional reaction in protein post-translational modifications, p53 ubiquitination can be reversed by deubiquitination, a reaction commonly catalyzed by ubiquitin-specific proteases (USPs) (Kwon et al., 2017). Among several USPs that are known to target p53, USP7 or HAUSP is one of the relatively well-characterized USPs that critically contribute to the regulation of p53 stability (Lim et al., 2004). Of interest is that HAUSP can also target MDM2 for deubiquitination representing a complex mechanism of p53 regulation (Li et al., 2004). Ubiquitination of p53 is often associated with its nuclear export to the cytoplasm (Boyd et al., 2000), where the ubiquitinated p53 is recognized as a substrate for degradation by proteasome resulting in a decrease in p53 abundance. However, under certain conditions where the proteasome activity is hampered, p53 may accumulate in the cytoplasm.

Nonetheless, p53 cytoplasmic distribution prevents it from binding to DNA, equivalent to functional p53 inactivation. Indeed, cytoplasmic p53 accumulation is found in a subset of human cancers (Lu et al., 2000). While the proteasome is primarily the place for p53 protein turnover, the autophagy-lysosome machinery has also been reported to participate in regulating p53 levels. However, the contribution of the autophagy-lysosome axis to p53 degradation seems to limit mutant p53 in a context-dependent manner (Xu et al., 2021).

In addition to the change in the p53 protein abundance, p53 activity can also be regulated via post-translational modifications. For example, by counteracting against p53 acetylation, which is necessary for its transcription activity, deacetylation of p53 by HDAC such as histone deacetylase eight diminishes p53 transcription activity (Qi et al., 2015). Another type of post-translational modification is protein methylation, which was also reported to be one of the mechanisms of p53 regulation. For instance, histone lysine methyltransferases KMT5 (Set9), KMT3C (Smyd2), and KMT5A (Set8) were reported to methylate p53 at specific C-terminal lysine residues. Thus, dependent on the site of modification, p53 methylation can either augment or attenuate p53 transcriptional activity. Furthermore, like acetylation/deacetylation, methylated lysine can be demethylated by the lysine-specific demethylase, such as KDM1 (LSD1), promoting p53 demethylation in interfering with the interaction of p53 with its co-activator 53BP1 and subsequent the induction of apoptosis (Scoumanne and Chen, 2008).

## The p53-Mediate Barrier to Cell Proliferation

It is well documented that p53 is typically growth inhibitory, or p53 is usually incompatible with increased cell proliferation. Based on this notion, induction of cell proliferation would predict a reduction in p53 activity/level. Zwang et al. reported that mitogen such as EGF-induced proliferation in normal human mammary epithelial cells was mediated by two temporally separable waves of growth signals during which induction of metabolic pathways is associated with downregulation of antiproliferative genes (Zwang et al., 2011). Many of the antiproliferative genes are the target genes of p53. Of interest is the finding that the second wave of growth signal drives cells passing the restriction point concurrent with p53 downregulation. To demonstrate the importance of p53, the authors used siRNA to knockdown p53 expression. Remarkably, p53 downregulation allowed cells to bypass the second wave of growth signals to cross the restriction point entering the S-phase. The study revealed that growth factor-induced cell proliferation must override a p53-dependent constraint, consistent with the notion that p53 functions as a barrier to cell proliferation (Vousden and Lane, 2007). Though the mechanism of EGF-induced p53 downregulation was not investigated, the authors showed that activation of PI3K and AKT was necessary to reduce the expression of p53-mediated antiproliferative genes. It has been shown that AKT can phosphorylate both MDM2 and MDMX, resulting in enhanced p53 ubiquitination/degradation, providing a plausible mechanism underlying the grow-promoting signal-induced p53 downregulation (Lopez-Pajares et al., 2008). In line with Zwang et al., Lei et al. also reported p53 downregulation in mitogen-induced cell proliferation (Lei et al., 2011; Zwang et al., 2011). The studies together implicate an essential role of basally expressed p53 in restraining cell proliferation. Pro-growth signals breach this growth constraint by stimulating MDM2/MDMX-mediated p53 turnover, promoting cell proliferation.

In agreement with the fundamental importance of metabolism in cell growth, induction of cell proliferation is contingent upon metabolic reprogramming from catabolic to anabolic metabolism. In accordance with its function in growth inhibition, p53 typically antagonizes anabolic pathways while stimulating oxidative phosphorylation. Available information indicates that p53 mainly regulates cellular metabolism in a transcription-dependent fashion (Vousden and Ryan, 2009). The basal p53-mediated restraint on cell proliferation would suggest a scenario in which basally expressed p53 could keep anabolic metabolism in check under the homeostatic condition. However, basal p53 typically possesses little transcription activity. Therefore, it is largely unknown whether and how p53 could regulate metabolism independent of its transactivation activity. An early study by Kawauchi et al. showed that loss of p53 either via gene knockout or siRNA-mediated knockdown was associated with induction of glycolysis (Kawauchi et al., 2008). Mechanistically, the authors demonstrated that p53 loss resulted in activation of NF-κB, which induced the expression of Glut3, promoting glycolytic metabolism. While the antagonistic interaction between p53 and NF-kB has been well documented, the study by Kawauchi et al. implicates that basal p53 can keep the NF-kB pathway under control, and a mere drop of p53 level would unrestraint its restriction unleashing NF-kB activity to promote anabolic metabolism (Kawauchi et al., 2009). Of note, Zwang et al. also reported that p53 downregulation was associated with induction of metabolic enzymes related to steroid, cholesterol, and lipid metabolism, whose intermediate products are critical substrates for cell division (Zwang et al., 2011).

The studies together suggest a model in which basally expressed p53 can keep anabolic metabolism in check to maintain homeostasis. In accordance, cell growth signals disable this p53-mediated metabolic constraint to induce anabolic metabolism, promoting cell proliferation. Therefore, further investigation is warranted to explore how basally expressed p53 keeps anabolic pathways under control.

## p53-Mediate Homeostatic Regulation of Immune and Inflammatory Response

Like cell proliferation that depends on anabolic metabolism, T cell activation represents another typical process involving a metabolic switch from catabolic to anabolic metabolism. Wang et al. demonstrated that metabolic reprogramming from the TCA cycle to the anabolic pathways, including glycolysis, pentose-phosphate, and glutaminolysis, is coupled with T cell activation (Wang et al., 2011). This switch to anabolic metabolism is necessary to meet the increased demands for the bioenergetic and biosynthesis as suppression of anabolic pathways genetically or pharmacologically blocked T cell activation. Mechanistic analysis revealed that the master transcription factor Myc is responsible for the increased glycolysis and glutaminolysis. Remarkably, a study by Watanabe et al. revealed that p53 downregulation is necessary for antigen-specific activation of T cell proliferation (Watanabe et al., 2014). While the authors did not examine the metabolic changes, increased, T cell proliferation is expected to be concurrent with metabolic reprogramming, which many studies have validated since the report by Wang et al. (Wang et al., 2011). Hence, it is conceivable to speculate that p53 downregulation enables the metabolic switch to anabolism to fuel T-cell proliferation, implicating an antagonistic interaction between p53 and Myc in the regulation of metabolism.

Studies have also uncovered an important role of p53 in B cell activation and expansion (Phan and Dalla-Favera, 2004). During the germinal center (GC) reaction in the lymph nodes, the activated B cells undergo cycles of expansion and specific genome remodeling, for instance, somatic hypermutations and class switch recombination. Highly expressed BCL6 in B cells within the GC is essential to regulate these events. BCL6 transcriptionally represses p53 expression (Phan and Dalla-Favera, 2004), which not only evades p53-dependent apoptosis but also allows B cell proliferation/expansion. Like T cells, B cell proliferation also relies on metabolic reprogramming, where mTOR and c-Myc-mediated glycolysis and anabolic metabolism were reported to contribute to B cell activation in the GC (Calado et al., 2012; Dominguez-Sola et al., 2012; Ersching et al., 2017). While the studies did not directly examine the interaction between p53 and c-Myc/mTOR, it is conceivable that p53 downregulation is conducive to the stimulation of c-Myc/mTOR (Feng et al., 2005). Further studies are warranted to address the antagonistic interactions.

The inflammatory response is energy-consuming process and relies on anabolic programs. For instance, in response to LPS stimulation, macrophages undergo metabolic reprogramming from oxidative phosphorylation to glycolysis via activation of the mTOR-HIF1α pathway (Covarrubias et al., 2015), resulting in induction of μPFK2 (Rodríguez-Prados et al., 2010) and GLUT1 (Freemerman et al., 2014). The production of IL1β is also contingent upon the activation of mTOR-HIF1α (Tannahill et al., 2013; Moon et al., 2015a) and fatty acid synthase (Moon et al., 2015b). The importance of p53 in inflammation was revealed with p53 knockout mice that exhibited inflammation so severe that some of the mice died from unresolved inflammation before the onset of tumorigenesis (Martínez-Cruz et al., 2009). Such a role of p53 in inflammation seems not unexpected considering the tight association of chronic inflammation with tumorigenesis (Gudkov and Komarova, 2016), though the underlying molecular details are still being actively investigated.

Macrophage is one of the major cell types that contribute to the inflammatory responses. Depending on stimuli, macrophages can be induced into different functional states, for instance, M1 or classically activated macrophages and M2 or activated macrophages, according to the simplified classification method. M1 macrophages are pro-inflammatory that is characterized by the release of inflammatory cytokines [IL-1β, IL-12, IL-6, and tumor necrosis factor (TNF)], reactive oxygen species (ROS), and nitrogen species, whereas M2 macrophages, in contrast, participate in the anti-inflammatory response to facilitate wound healing and tissue repair. Importantly, M1 and M2 are intimately linked to and controlled by distinct metabolic programs (Covarrubias et al., 2015). Stimulation of M1 polarization is associated with induction of glycolysis, fatty acid synthesis, amino acid metabolism, and inflammatory cytokines. The transcriptional program in M1 macrophage is primarily mediated by the mTOR-HIF-1α pathway (Covarrubias et al., 2015). M2 macrophages preferentially rely on β-oxidation of fatty acids and mitochondrial respiration for their sustenance and functional activation. Type 2 cytokines, such as IL-4 and IL-13, signal to activate the latent STAT6 transcription factor through their cognate receptors. STAT6 promotes the metabolic transition to oxidative metabolism by inducing genes essential in FAO and mitochondrial biogenesis. In addition, STAT6 transcriptionally induces PGC-1β, PPARγ, and PPARδ, which synergize with STAT6 to enhance the expression of alternative activation markers and stabilize the metabolic switch to oxidative metabolism.

In support of the role of p53 in inflammation, Li et al. reported that loss of p53 stimulated whereas activated p53 impeded M2 macrophage polarization (Li et al., 2015). Using a combination of genetic and pharmacological approaches, the authors demonstrated that p53 selectively inhibits M2 polarization by downregulating M2 gene expression. While the authors did not examine the metabolic changes associated with macrophage polarization, they demonstrated an antagonistic interaction between p53 and c-Myc involved in the regulation of M2 polarization. Specifically, p53 repressed the expression of Myc genes during M2 polarization. Given the well-established role of Myc in the control of anabolic metabolism, the results are consistent with the metabolic characteristics associated with M2 macrophages where glycolysis is downregulated whereas mitochondrial respiration is upregulated (Phan et al., 2017).

In tumorigenesis, tumor cells can substantially impact surrounding cells to shape the tumor microenvironment (TME) that promotes cancer progression. The dynamic interactions between tumor cells and immune cells have been widely reported. However, how p53 participates in regulating the tumor immune microenvironment is only beginning to be investigated. A recent study by Wang et al. showed that implanted mammary carcinoma cells acted on their surroundings in the host to induce an immunosuppressive microenvironment facilitating tumor growth (Wang et al., 2020). A contribution of p53 to the regulation of the immune microenvironment was demonstrated with a genetically engineered mouse model expressing a phospho-resistant MDMX. A prior study identified the 314-serine residue of MDMX as the phosphorylation site by receptor tyrosine kinases as well as the stress kinase p38. MDMX-S314 phosphorylation stabilized the MDM2/MDMX complex leading to augmented p53 degradation (de Polo et al., 2017). To investigate the effect of tumor cells on the p53 pathway in surrounding cells, the authors implanted an EO77 mammary carcinoma cell line that harbors mutant p53 into syngeneic host mice expressing wild-type p53. The implanted tumor cells imposed marked influence on the neighboring cells, evidenced by reduced p53 abundance in peritumor cells. This effect of the implanted tumor on peritumor cells appeared to be mediated by MDMX-S314 phosphorylation as the p53 decline in mice expressing *Mdmx*S314 A was blocked. Of significance were the observations that impediment of p53 decline was associated with mitigation of the suppression of immune responses as reflected by increased immune cell tumor infiltration and enhanced macrophage M1 polarization compared with that in wild-type mice.

Moreover, the improved immune response in *Mdmx*S314 A mice was coupled with a significant delay in tumor growth. Thus, the study implicates that tumor cells can induce an immune suppressive microenvironment by downregulating p53 in peritumor cells, suggesting a role of basal p53 in the maintenance of the immune response, However, further studies will be necessary to understand how basally expressed p53 preserves immune homeostasis.

Within the context of the tissue microenvironment, p53 was reported to play a role in maternal reproduction by controlling the expression of basal as well as inducible level of leukemia inhibitory factor (LIF), a cytokine critical for implantation (Hu et al., 2007).

## p53-Mediated Homeostatic Regulation of Cell Competition

Within tissues, cell-cell interactions are regulated by a host of mechanisms to preserve homeostasis. In addition to cell-intrinsic mechanisms to eliminate cells that contain unrepaired damages or are suboptimal, cells can also sense their neighbors to determine relative fitness, which constitutes an important mechanism to eliminate comparatively weaker cells, a process described as cell competition. Ample evidence indicates that cell competition is involved in various processes such as development, tissue homeostasis, and tumorigenesis. Cell competition to eliminate damaged and unhealthy cells are expected to yield positive and beneficial outcomes. It is, however, also conceivable that competition may contribute to tumor development. In accordance, studies have shown that malignant cells acquire various mutations to gain growth advantages in competition with neighboring normal cells (Vishwakarma and Piddini, 2020). While diverse mechanisms of cell competition have been reported, one of the widely observed pathways involves increased levels of Myc (Paglia et al., 2020). Myc is an important determinant of relative cell fitness, with winner cells having higher Myc levels than losers. However, despite these advances, the precise mechanism Myc affects cell fitness is not fully understood.

Given the homeostatic function of p53 and the well-established role of cell competition in preserving tissue homeostasis, it is probably not unexpected that p53 has been reported in the regulation of cell competition. Bondar et al. reported that a moderate increase in p53 induced by treatment with radiation at a low dose of 1Gy was associated with a loser phenotype in hematopoietic stem and progenitor cells (HSPCs) (Bondar and Medzhitov, 2010). The authors compared the ability of HSPCs with radiation-induced higher p53 levels versus non-irradiated controls to repopulate the chimeric bone marrow. The HSPCs with higher p53 levels were outcompeted by untreated HSPCs resulting in a marked reduction of p53 expressing HSPCs. In line with p53-mediated senescent function, high p53 expressing HSPCs were eliminated via the senescent program.

Like the modest p53 induction by treatment with low-dose radiation, a genetic method-induced mild increase in p53 also resulted in a less competitive status in both embryos and adult cells. Zhang et al. generated haploinsufficiency of Mdm2 and Mdm4 mice where p53 was slightly elevated but had little effect on growth (Zhang et al., 2017). However, mosaic haploinsufficiency of these genes rendered the cells with a competitive disadvantage during embryogenesis in mosaic embryos and adult tissues with active cell proliferation such as bone marrow, spleen, and testis. Of interest is the observation that the competitive disadvantage due to a mild increase in p53 levels was associated with reduced cell proliferation only in the developmental embryos but not in adult tissues, indicative of a context-dependent mechanism behind cell competition.

The finding that a moderate increase in p53 resulted in a less fit status would predict that reduced p53 level/activity might be associated with a more fit status. Indeed, in a study of embryonic development, knockdown p53 rendered embryonic stem (ES) cells a competitive advantage resulting in the replacement of wild-type ES cells when they were co-injected into the mouse embryo (Dejosez et al., 2013). While the study did not investigate how p53 downregulation could provide a competitive advantage, a recent study in mouse embryogenesis uncovered a novel mechanism of p53-mediated control of mTOR (Bowling et al., 2018). The authors demonstrated mTOR as a crucial determinant for cell competition during the early post-implantation stages. Higher mTOR activity provided a competitive advantage, whereas lower mTOR activity resulted in a disadvantage in competition. Of interest is the finding that p53 acted upon mTOR to control the activity of this metabolic enzyme. While elevated p53 repressed mTOR, reduced p53 expression by knockdown was associated with enhanced mTOR activity resulting in a marked increase in the competitive advantage. With the well-established metabolic function of mTOR, the study revealed a novel mTOR-dependent metabolic mechanism behind cell competition. Numerous studies have shown an antagonistic interaction between p53 and mTOR (Feng et al., 2005). For instance, p53 was reported to suppress mTOR activity by activating SESTRIN gene expression (Budanov and Karin, 2008) inducing the levels of REDD1 (Brugarolas et al., 2004). Further investigation is necessary to interrogate the functional interaction between basal p53 and the mTOR pathway.

## p53-Mediated Homeostatic Regulation of Stem Cell Self-Renewal and Differentiation

The p53-mediated homeostatic function also contributes to maintaining the balance between self-renewal and differentiation of stem cells (Jain et al., 2012). The early observation that in contrast with somatic cells, p53 is expressed at relatively high levels in mouse embryos or mouse embryonic stem cells (ESCs) (Schmid et al., 1991) suggests that p53 might function in early development and cell differentiation. However, subsequent studies revealed that the elevated p53 level in ESCs does not result in apoptosis or differentiation, primarily due to its cytoplasmic distribution. The subcellular p53 localization in ESCs was shown to be regulated by SIRT1-mediated deacetylation (Han et al., 2008) and might also be by MDM2/MDMX-mediated ubiquitination (Menéndez et al., 2011)*.* The high level of p53 in the cytoplasm may keep it poised in response to potential stress. Indeed, DNA damage triggered by X-ray or UV irradiation induces p53 redistribution to the nucleus leading to p53 activation and subsequent induction of p53 target genes that promote ESCs differentiation (Lin et al., 2005)*.* The available information supports the essential role of p53 in regulating the balance between pluripotency and differentiation in ESCs.

Similar to ESCs, p53 has been implicated in regulating induced pluripotent stem cells (iPSCs), which can be established by introducing reprogramming factors (OCT4, SOX2, KLF4, and c-MYC) into somatic cells. iPSCs display the ability of self-renewal and differentiation into many cell types, a feature like embryonic stem cells. Somatic cells undergo transitions in gene expression profile, epigenetic status, metabolic characteristics, and cellular morphology (Folmes et al., 2012). Ample evidence indicates that p53 functions as a barrier to somatic cell de-differentiation or reprogramming. Indeed, a recent study by Zhao et al. demonstrated that siRNA-mediated knockdown of p53 in human adult fibroblasts enhances iPS cell induction efficiency up to 100-fold (Zhao et al., 2008; Mathieu et al., 2014).

In line with the p53-mediated barrier function, the function of p53 in iPS is suppressed usually via a mode of post-translational modifications, which include ubiquitylation, acetylation, phosphorylation, methylation, or sumoylation of specific residues of p53 (Jain et al., 2012). Lee et al. reported that Aurora kinase A phosphorylates p53 (at Ser212 and Ser312) during iPS reprogramming, inhibiting p53 activity (Lee et al., 2012). Another study reported that Aurora kinase A-mediated p53 phosphorylation at Ser315 promoted MDM2-dependent ubiquitination and degradation of p53 protein (Katayama et al., 2004).

An additional type of modification frequently involved in the regulation of p53 in human ES cells is the acetylation of a lysine residue in the p53 protein. It was reported that despite being distributed in the nucleus of human ES cells, p53 is transcriptionally inactive because the 120/373 lysine residues are not acetylated. Although Sirt1 can maintain the non-acetylated status, a NAD-dependent deacetylase induced transcriptionally by Oct4 (Zhang et al., 2014), some lysine residues in the p53 protein can also be methylated, which often results in suppression of p53 transcription activity. Thus, it is conceivable that acetylation of certain lysine residues in the p53 protein is necessary for its transcription activity; methylation of the identical lysine residues would prevent their acetylation leading to p53 inactivation (Berger, 2010). Interestingly, preventing lysine methylation by replacing it with arginine at K370 R or K382 R resulted in p53 activation (Zhu et al., 2016), suggesting that p53 methylation-mediated p53 repression is not merely competing with activating acetylation.

Thus, it is clear that p53 activity is attenuated or inactivated in stem cells, which appears necessary to allow stem cells to replicate. The inactivation of p53 in stem cells can result from either a deficiency in p53 transcriptional activity or post-translational modifications on the p53 protein that result in an inactive p53 protein. Collectively, these studies suggest that p53 controls the transition between cell self-renewal and differentiation. p53 restricts the ability of somatic cells to undergo reprogramming into iPSCs.

The importance of metabolic regulation during the reprogramming to pluripotency has been well documented (Mathieu et al., 2014). Relative to their somatic counterparts, pluripotent stem cells, including ESCs and iPSCs, exhibit a high rate of glycolysis similar to aerobic glycolysis in cancer cells, which is necessary for maintaining stemness. This unique glycolytic metabolism in ESCs and iPSCs can provide bioenergetic supplies and promote the pentose phosphate pathway crucial for preserving redox homeostasis. Somatic cells undergo a metabolic switch from oxidative phosphorylation to glycolysis during reprogramming, which elicits the initiation and progression of reprogramming to iPSCs.

Ample evidence has shown that there is a very dynamic cross-talk between metabolic pathways and epigenetic programs. Cells continuously modify their metabolic programs and activities in response to nutrient availability, extracellular signals, and reprogramming/differentiation cues. Many intermediary metabolites can function as cofactors for epigenetic enzymes that catalyze histone methylation and acetylation reactions, contributing to the regulation of gene transcription. This cross-talk between intermediary metabolism and epigenetics has been demonstrated as central mechanisms by which metabolic pathways are engaged in stem cell fate determination (Kaelin and McKnight, 2013). Pluripotent stem cells are featured with bivalent chromatin regions, which encompass activating histone modifications, such as histone H3 lysine four trimethylations (H3K4me3), and repressive modifications histone H3 lysine 27 trimethylation (H3K27me3). Such bivalent chromatin domains enable developmental genes to maintain their repressive status without differentiation signals while allowing immediate activation in response to signal cues. Evidence indicates that epigenetic regulation of self-renewal and differentiation are intimately interfaced with cellular metabolism (Kaelin and McKnight, 2013). For instance, H3K4me3 is regulated by SAM levels generated through one-carbon metabolism (Shyh-Chang et al., 2013; Shiraki et al., 2014). Repressive H3K9me3 and H3K27me3 marks are regulated in an αKG-dependent manner through demethylation by JmjC-domain containing histone demethylases (JHDMs) and ten-eleven translocation (TET) enzymes (Kaelin and McKnight, 2013). With abundant evidence indicating an important role of p53 in regulating the balance between pluripotency and differentiation in stem cells, it will be interesting to link the p53 status to the metabolic regulation of stem cell fate. The correlation of reduced p53 nuclear abundance with glycolytic metabolism in stem cells is in line with the anti-glycolytic function of p53. Further studies will be necessary to understand better how a decrease in nuclear p53 abundance/activity can regulate metabolic pathways and the cross-talk with the epigenetic programs in stem cells.

## The p53-Mediated Homeostatic Function in Stress Response

A proper stress response is critical for maintaining homeostasis. When encountered with different levels of stress, cells have to determine the fate between survival and death. In response to excessive stress that is destructive to genome integrity and other cellular structures, cells must sense the intensity of damage and rapidly activate responses such as cell cycle arrest, DNA damage repair, senescence, or apoptosis if the damage is unrepairable. However, living cells or organisms are often exposed to temporary and low levels of stress in our daily lives. In response to such transient and mild stress, inciting cellular senescence or cell death would not make sense economically. Under such conditions, cells must finely tune their response to the perturbation based on stress level. Abundant evidence indicates that p53 is one of the key players in regulating the stress response (Kruiswijk et al., 2015). The importance of p53 in mediating cellular response to severe stress has been extensively investigated and relatively well understood. For instance, p53 is highly responsive to harsh conditions such as DNA damage, which activates p53 transcriptional activity, resulting in upregulation of genes whose products induce senescence or apoptosis to eliminate damaged cells. Relative to its contribution to defending organismal integrity under severe stress conditions, how p53 regulates responses to mild stress is not well studied and remains incompletely understood.

Available information indicates that mild stress can induce an adaptive response, an evolutionally conserved defense mechanism to preserve homeostasis (Calabrese et al., 2016). Evidence reveals that cellular adaptation to mild stress is an active process mediated by anabolic metabolism, which is critical in supporting cell viability and fueling the biosynthesis of biomolecules to mount the defense (Wang et al., 2019). While p53 was reported to be involved in the adaptive response (Horie et al., 2002; Lall et al., 2014), the underlying mechanisms are only beginning to be investigated. With the well-documented role of p53 in promoting oxidative phosphorylation while suppressing glycolysis, the anabolism-mediated adaptive response would suggest a compromised p53 activity. Indeed, it was reported that low-dose radiation-induced adaptive and protective response is associated with p53 downregulation (Lall et al., 2014), in line with p53’s pro-death function. Of interest is that concurrent with low-dose radiation induced p53 downregulation is the upregulation of HIF1α and consequent induction of glycolysis and the pentose phosphate pathway. The study further showed that a low-dose radiation-induced metabolic switch is required for the protective adaptive response, consistent with an anabolism-dependent mechanism behind the adaptive stress response (Wang et al., 2019). Likewise, the low-dose arsenic-induced protective response is also associated with stimulation of metabolic reprogramming from oxidative phosphorylation to glycolysis, which is similarly mediated by p53 decline concurrent with however induction of NF-κB, which is known to induce the expression of several glycolytic genes (Ganapathy et al., 2014). The results suggest that basal p53 could keep anabolic metabolism in check and the downregulation of basal p53 becomes conducive for the induction of anabolic pathways. Given the critical contribution of HIF1α and NF-κB to the control of anabolic metabolism, the antagonistic interaction between p53 and NF-κB (Ak and Levine, 2010), or p53 and HIF1α (Obacz et al., 2013) may represent an important mechanism for the metabolic regulation of the adaptive stress response, though the precise mechanisms by which basal p53 restrains HIF1α and NF-κB remain to be elucidated.

Studies have shown that the adaptive stress response is primarily mediated by a modest increase in ROS as treatment of cells with an antioxidant such as N-acetyl cysteine could mainly diminish the adaptive response (Ganapathy et al., 2014). The role of p53 in oxidative stress is well known. However, most studies have shown p53 activation by ROS (Kruiswijk et al., 2015). Evidence indicates that ROS-induced response depends on the level of ROS and the duration (Finkel 2012). Exposure to high levels of ROS and long durations can cause damage to DNA, RNA, or protein, whereas a transient increase of a modest amount of ROS would function as signal cues to induce a cellular response. It is conceivable that contrary to the high level of ROS that activates p53, a low level of ROS may stimulate signal pathways leading to p53 downregulation. ROS is known to primarily react with cysteine residues within target proteins, particularly low-pKa cysteine residues commonly found at the reactive site of enzymes (Finkel 2012). Protein phosphatases are well known extremely sensitive to be inactivated by ROS, resulting in activation of their target protein kinases (Finkel 2012). It was reported that protein kinases could downregulate p53 by phosphorylating MDMX increasing the MDM2/MDMX complex (Gerarduzzi et al., 2016; de Polo et al., 2017). Within the context of cellular metabolism, the association of anabolism-mediated adaptive stress response with p53 decline seems in line with p53-mediated repression of anabolic metabolism (Vousden and Ryan, 2009). Of note, the adaptive stress response can be beneficial when transient, however, persistent or chronic stress is usually associated with homeostatic imbalance, leading to pathological outcomes. While multiple factors might be involved, a sustained p53 downregulation during prolonged stress would likely contribute to the disruption of homeostasis and whereby development of diseases.

## Conclusion

**FIGURE 1:**
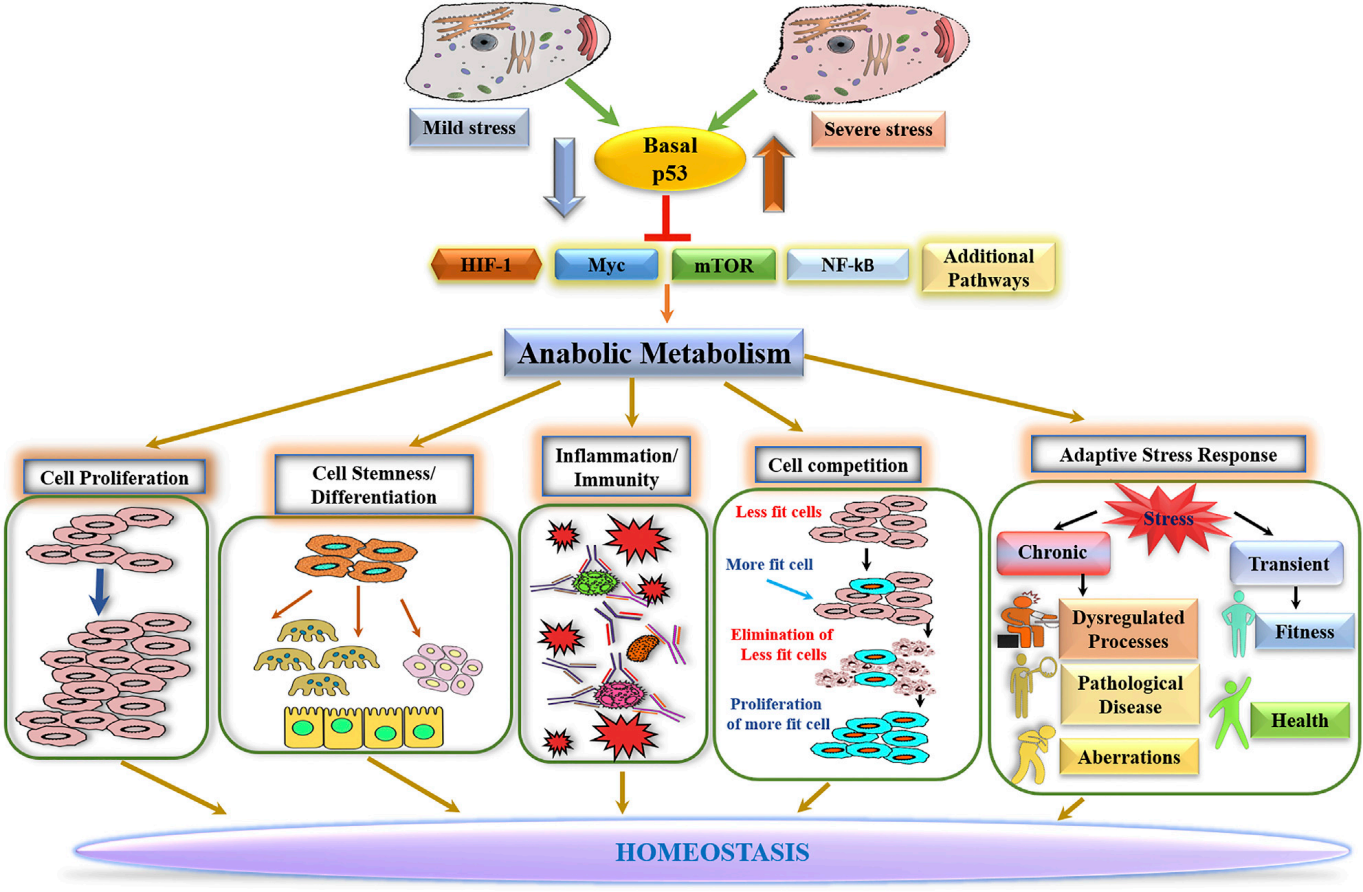
Basal p53-mediated homeostasis.

Homeostasis, a property crucial for normal physiology, is maintained by coordinated actions of diverse cellular processes and pathways. As a process fundamental to all biological functions, metabolism is intimately involved in regulating every facet of biological processes, which contributes to maintaining homeostasis. The basally expressed p53 safeguards homeostasis by keeping anabolic metabolism in check, which functions as a barrier to cell proliferation and governs numerous anabolism-dependent processes. In line with this notion are the observations that induction of many anabolism-driven processes is accompanied by a decline in nuclear p53 level/activity. While the ability of p53 to antagonize against Myc, HIF1α, NF-κB, or mTOR likely contributes to restraining anabolic metabolism (Figure 1), p53-mediated maintenance of metabolic homeostasis might involve a coordinated interaction of diverse processes at the systems level. Indeed, a recent study with genetically engineered mouse models revealed a high degree of connectivity between p53 and process-specific transcription factors (Mak et al., 2017). Of note is that most of the genes whose protein products are the key regulators and enzymes of metabolic pathways are extremely sensitive to changes in p53 protein levels, implicating that alterations in p53 abundance/activity may have very broad effects on metabolic programs. Further investigation is warranted to dissect the p53 network at the systems level to understand p53-mediated homeostatic function better.
